# Localization of BDNF and Calretinin in Olfactory Epithelium and Taste Buds of Zebrafish (*Danio rerio*)

**DOI:** 10.3390/ijms23094696

**Published:** 2022-04-23

**Authors:** Marialuisa Aragona, Caterina Porcino, Maria Cristina Guerrera, Giuseppe Montalbano, Rosaria Laurà, Maria Levanti, Francesco Abbate, Teresa Cobo, Gabriel Capitelli, Fabrizio Calapai, José A Vega, Antonino Germanà

**Affiliations:** 1 Zebrafish Neuromorphology Lab, Department of Veterinary Sciences, University of Messina, 98168 Messina, Italy; mlaragona@unime.it (M.A.); catporcino@unime.it (C.P.); mguerrera@unime.it (M.C.G.); gmontalbano@unime.it (G.M.); laurar@unime.it (R.L.); mblevanti@unime.it (M.L.); abbatef@unime.it (F.A.); 2 Departamento de Cirugía y Especialidades Médico-Quirúrgicas, Universidad de Oviedo, 33006 Oviedo, Spain; tcobo@uniovi.es; 3 Faculty of Medical Sciences, University of Buenos Aires, Viamonte 1053, CABA, Buenos Aires 1056, Argentina; gcapitelli@rec.uba.ar; 4 Department of Chemical, Biological, Pharmaceutical and Environmental Sciences, University of Messina, Viale F. Stagno d’Alcontres 31, I-98166 Messina, Italy; fabrizio.calapai@unime.it; 5 Grupo SINPOS, Universidad de Oviedo, 33003 Oviedo, Spain; javega@uniovi.es; 6 Departamento de Morfología y Biología Celular, Universidad de Oviedo, 33006 Oviedo, Spain; 7 Facultad de Ciencias de la Salud, Universidad Autónoma de Chile, Santiago 7500912, Chile

**Keywords:** BDNF, chemosensory organs, olfactory epithelium, taste buds, isolated cutaneous chemosensory cells, development, zebrafish

## Abstract

Brain-derived neurotrophic factor (BDNF) is a member of the neurotrophin family and it is involved in several fundamental functions in the central and peripheral nervous systems, and in sensory organs. BDNF regulates the chemosensory systems of mammals and is consistently expressed in those organs. In zebrafish, the key role of BDNF in the biology of the hair cells of the inner ear and lateral line system has recently been demonstrated. However, only some information is available about its occurrence in the olfactory epithelium, taste buds, and cutaneous isolated chemosensory cells. Therefore, this study was undertaken to analyze the involvement of BDNF in the chemosensory organs of zebrafish during the larval and adult stages. To identify cells displaying BDNF, we compared the cellular pattern of BDNF-displaying cells with those immunoreactive for calretinin and S100 protein. Our results demonstrate the localization of BDNF in the sensory part of the olfactory epithelium, mainly in the ciliated olfactory sensory neurons in larvae and adult zebrafish. Intense immunoreaction for BDNF was also observed in the chemosensory cells of oral and cutaneous taste buds. Moreover, a subpopulation of olfactory sensory neurons and chemosensory cells of olfactory rosette and taste bud, respectively, showed marked immunopositivity for calcium-binding protein S100 and calretinin. These results demonstrate the possible role of BDNF in the development and maintenance of olfactory sensory neurons and sensory cells in the olfactory epithelium and taste organs of zebrafish during all stages of development.

## 1. Introduction

Brain-derived neurotrophic factor (BDNF) and its high-affinity signaling receptor TrkB are the most widely investigated parts of the neurotrophin family. Neurotrophins (NTs) are a family of growth factors promoting the development, survival, and phenotypic differentiation of discrete neuronal populations [1]. Furthermore, they regulate heterogeneous functions in some non-neuronal tissues, acting throughout specific transmembrane receptors with tyrosine kinase activity (Trk) [2,3]. Both NTS and their signaling receptors are evolutionarily conserved among vertebrates [4,5,6,7,8], including fish [9,10,11,12]. In particular, the gene encoding for BDNF in zebrafish is about 90% identical to its mammalian counterpart [13], and its respective protein was found in developing and adult fish [14,15]. The BDNF/TrkB system is expressed in the brain [16,17,18,19], cranial nerves [20], and sensory systems and some organs such as the inner ear [21,22], lateral line system (LLS) [11,23,24], and retinas [25,26]. Nevertheless, although TrkB was detected in fish taste buds [27], no data are available about the occurrence of BDNF in these organs. Furthermore, it is unknown whether BDNF and TrkB, alone or coexpressed, are present in the olfactory rosette and isolated cutaneous chemosensory cells (ICCC) of zebrafish. The distribution and structure of taste buds [28] and the olfactory epithelium [29] are well-known in developing or adult zebrafish, and became an attractive and widely employed model in experimental embryology and cell biology studies [30]. This model was also used in experiments for odor detection [31], hearing, and deafness [32]. Conversely, isolated cutaneous chemosensory cells are poorly understood, and whether they are under the control of the BDNF/TrkB system has not been investigated. So, exploring the localization of BDNF in chemosensory organs could help in clarifying its function within them. The present study investigates the distribution of BDNF in developing and adult chemosensory organs of zebrafish. Since those organs consist of heterogeneous cell populations, we attempted to identify cells displaying BDNF by comparing them with previous well-known markers such as calretinin, S100 protein, or TRPV4 [33,34,35].

## 2. Results

### 2.1. Localization of BDNF in the Olfactory Epithelium of Zebrafish during Development

**Figure 1:**
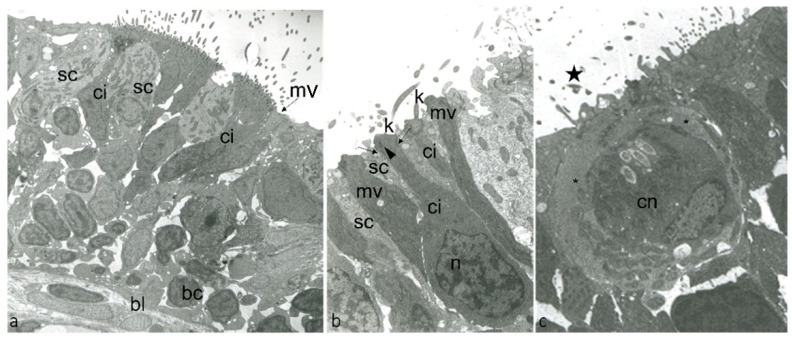
Transmission electron micrographs of ultrathin sections showing the surface of olfactory epithelium adult zebrafish: (**a**) ci, ONR ciliated; mv, ONR microvillous; sc, supporting cells; bc, basal cells; bl, basal lamina. (**b**) ci, ONR ciliated; n,“checkerboard” nucleus of ciONR; K, kinocilia**;** mv, ONR microvillous; sc, supporting cells; kinocilium basal body foot (arrowhead), junctional complex with neighboring supporting cells (arrows). (**c**) cn, crypt neurons with several cilia within the crypt (star); special supporting cells (*).

Zebrafish developed sensory mechanisms to detect and process essential signals for survival, feeding, and reproduction. Among them are senses of smell, taste, and the ICCC revealing chemical compounds and ions in water. The olfactory organ is localized in the dorsal part of the snout inside the olfactory cavity, connected with the aquatic environment formed by an olfactory epithelium organized in lamellae converging in a central raphe. The olfactory epithelium is a pseudostratified and columnar, and mainly constituted by bipolar neurons called olfactory sensory neurons (OSNs) and nonsensory cells (supporting and basal cells) (Figure 1a). In zebrafish, three main types of OSNs, ciliated, microvillous (Figure 1b), and crypt (Figure 1c), were identified.

**Figure 2:**
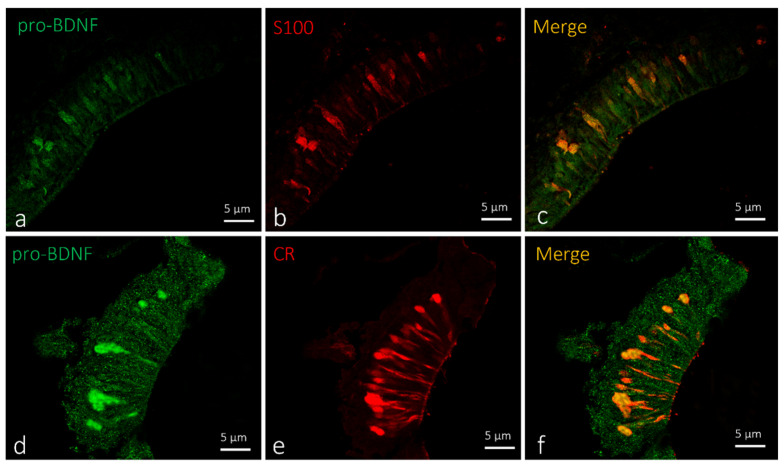
Olfactory lamellae of zebrafish larvae 5dpf, cross view. (**a**–**c**) Immunohistochemical detection (immunofluorescence method) of pro-BDNF and S100 protein. (**d**–**f**) Immunohistochemical detection (immunofluorescence method) of pro-BDNF and calretinin (CR). Subpopulation of olfactory epithelium sensory cells with an elongated shape showing immunoreactivity to (**a**,**d**) pro-BDNF, (**b**) S100 protein, and (**e**) calretinin. (**c**,**f**). Colocalization view. Magnification, 63×. Scale bar, 5 µm.

In zebrafish larvae, pro-BDNF was localized in a subpopulation of sensory cells in the olfactory epithelium with an elongated shape (Figure 2c,f). Therefore, to ascertain the exact nature of the cells displaying pro-BDNF, double immunofluorescence against S100 protein and calretinin was carried out. According to previous studies, calretinin and the S100 protein can immunomark different subpopulations of sensory cells in the olfactory epithelium [36,37]. Obtained results showed the colocalization of a subpopulation of sensory immunopositive cells for pro-BDNF with S100 protein (Figure 2c) and calretinin (Figure 2f).

**Figure 3:**
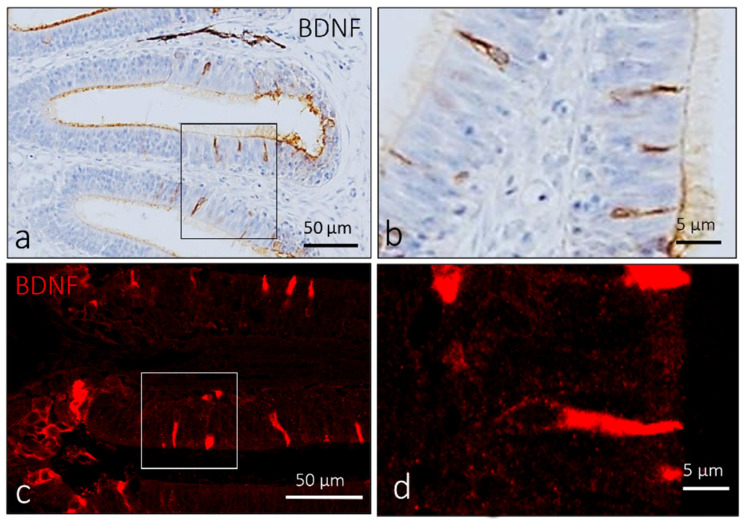
Olfactory lamellae of adult zebrafish, dorsal view. Immunohistochemical detection of BDNF: (**a**,**b**) peroxidase method, (**c**,**d**) immunofluorescence method. (**a**–**d**) Elongated sensory cells in sensory segment of olfactory lamellae show immunoreactivity to BDNF. (**a**,**c**) Scale bar 50 µm. (**b**,**d**) Scale bar 5 µm.

In the olfactory rosette of adult zebrafish, a subpopulation of elongated sensory cells showed intense immunoreaction for BDNF (Figure 3b). The immunoreaction was localized in the cytoplasm and ciliary processes of the apex (Figure 3c).

**Figure 4:**
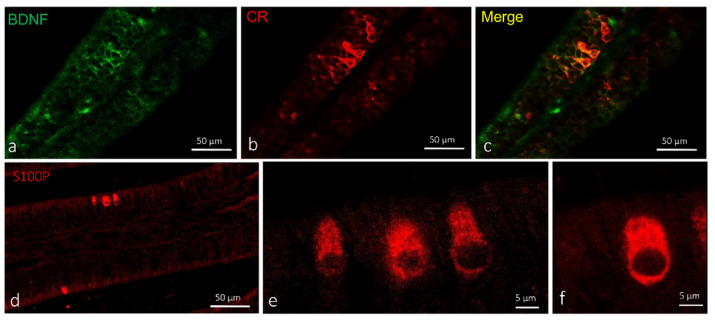
Olfactory lamellae of adult zebrafish, dorsal view. (**a**–**c**) Immunohistochemical detection (immunofluorescence method) of BDNF and calretinin. (**d**–**f**) Immunohistochemical detection (immunofluorescence method) of S100 protein. Ciliated sensory olfactory neurons in sensory segment of olfactory lamellae showing immunoreactivity to (**a**) BDNF and (**b**) calretinin. (**c**) Colocalization view (**d**–**f**). Crypt olfactory neurons showing immunoreactivity to S100 protein. Scale bar (**a**–**d**) 50 µm and (**e**,**f**) 5 µm.

Using double immunofluorescence, these cells were also positive for calretinin (Figure 4a–c) but not for S100 protein, found only in crypt olfactory neurons (Figure 4d–f). Therefore, on the basis of colocalization results, anatomical features, and ultrastructural investigation using the transmission electron microscope, these cells were identified as ciliated sensory olfactory neurons.

### 2.2. Localization of BDNF in Taste Buds and ICCC of Zebrafish during Development

**Figure 5:**
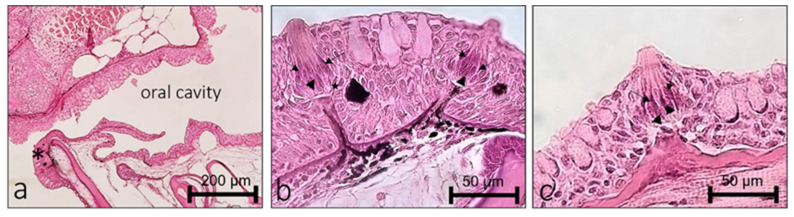
Light micrographs (hematoxylin/eosin staining) of adult zebrafish taste buds, dorsal view. (**a**) Oral cavity with taste buds, taste buds of the lips (*). (**a**,**b**)Taste buds showing light cells (arrowhead) add dark cells (arrow); (**b**) Merkel-like basal cells (star). Scale bar (**a**) 200 µm and (**b**,**c**) 50 µm.

Taste buds are chemosensory organs for detecting and evaluating environmental chemical stimuli distributed on the outer surface of the skin, head, lips, and oral cavity (Figure 5a). Mature taste buds are pear-shaped intraepithelial sensory organs located on a small dermal papilla. Two principal populations of sensory cells can be distinguished in taste buds: dark and light cells. The former are characterized by an apex with short microvilli, while the latter show a single large microvillus at the apex. Between sensory cells and the basal lamina, Merkel-like basal cells were described (Figure 5b,c).

**Figure 6:**
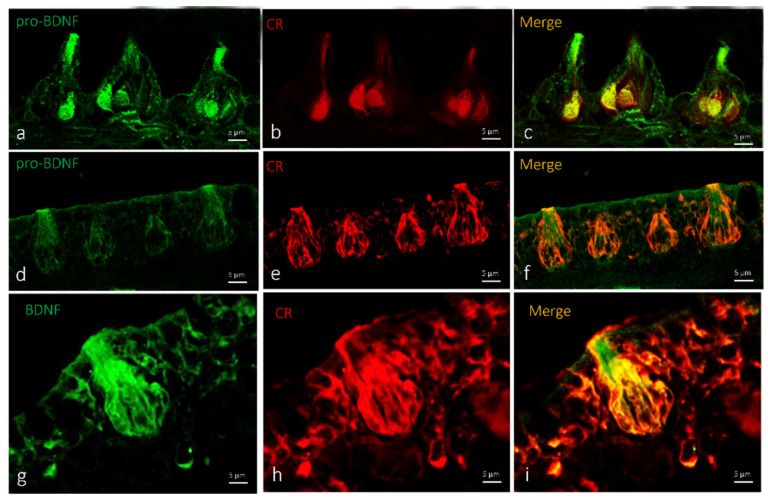
Cutaneous taste buds: (**a**–**c**) zebrafish larvae (5 dpf); (**d**–**i**) adult zebrafish, transversal view. (**a**–**c**) Immunohistochemical detection (immunofluorescence method) of pro-BDNF and calretinin. (**d**–**i**) Immunohistochemical detection (immunofluorescence method) of BDNF and calretinin. (**a**–**c**) Cytoplasmatic immunoreactivity for pro-BDNF and calretinin in sensory cells of cutaneous taste buds of zebrafish larvae (5 dpf). (**d**–**i**) Sensory cells of cutaneous taste buds of adult zebrafish showing immunopositivity to BDNF and calretinin. (**c**,**f**,**i**) Colocalization view. Scale bar, 5 µm.

**Figure 7:**
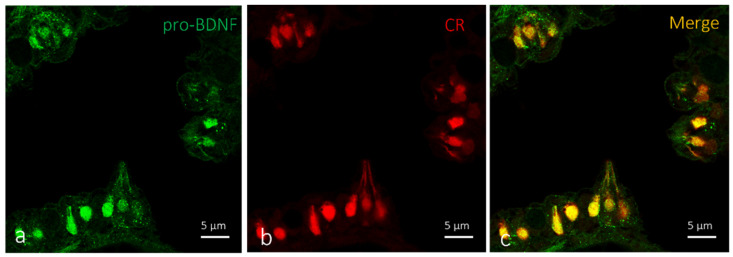

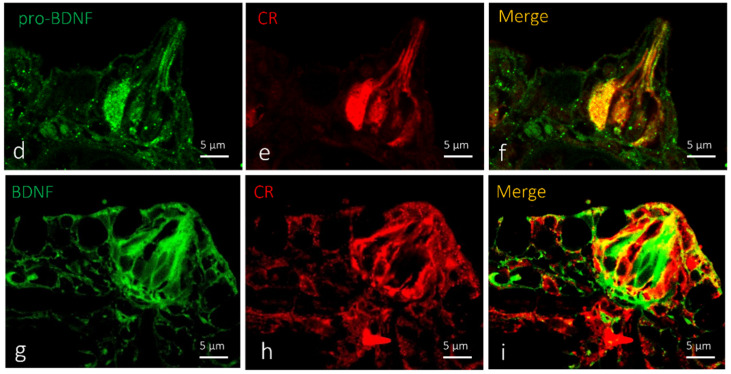
Oral taste buds: (**a**–**f**) zebrafish larvae (5 dpf); (**g**–**i**) adult zebrafish, transversal view. Immunohistochemical detection (immunofluorescence method) of (**a**–**f**) pro-BDNF and calretinin and (**g**–**i**) BDNF and dalretinin. Subpopulation of sensory cells that might be identified as light cells showing immunopositivity to (**a**,**d**) pro-BDNF and (**b**,**e**) calretinin. (**g**) BDNF in light sensory cells and (**h**) calretinin in dark sensory cells. (**c**,**f**,**i**) Colocalization view. Scale bar, 5 µm.

Single and double immunofluorescence were carried out on serial sections to localize BDNF. Results showed positive immunoreaction for BDNF in the cytoplasm of sensory cells in the taste buds of larvae and adult zebrafish. (Figure 6a,d,g and Figure 7a,d,g). Moreover, we found an identical immunoreaction distribution pattern for BDNF and calretinin in all investigated cutaneous taste buds (Figure 6). Cell types expressing BDNF were identified using a histological and morphotopographical approach, and colocalization with calretinin, which is considered to be a specific marker for the chemosensory cells of a zebrafish taste bud [33]. Using the double immunofluorescence method demonstrated that calretinin and BDNF are colocalized (Figure 6c,f,i) into the cutaneous taste buds’ sensory cells both in larvae (Figure 6b) and adults (Figure 6e,h). This evidence demonstrates the chemosensory origin of these cells.

In oral taste buds, BDNF is localized in the Merkel cells and cytoplasm of a subpopulation of sensory cells that might be identified as light cells (Figure 7a,d,g). The double immunoreaction demonstrated an overlap of pro-BDNF and calretinin immunopositivity in larval oral taste buds (Figure 7c,f). Moderate colocalization of the employed antibodies was observed, with a prevalence of the localization of calretinin (Figure 7h) in dark sensory cells, and BDNF in light sensory cells (Figure 7i).

**Figure 8:**
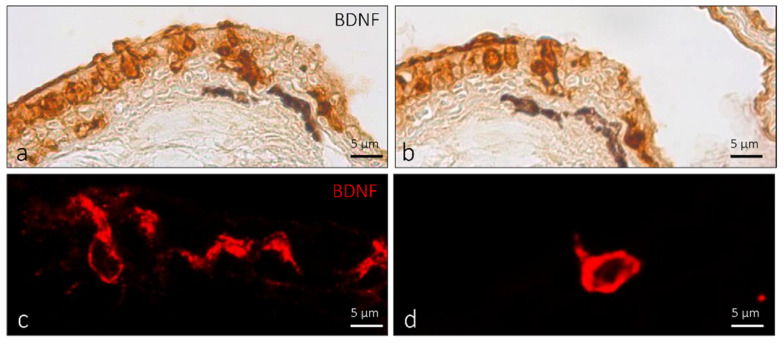
ICCC of adult zebrafish, dorsal view. Immunohistochemical detection of BDNF: (**a**,**b**) peroxidase method; (**c**,**d**) immunofluorescence method. (**a**–**d**) ICCC immunoreactivity to BDNF. Scale bar, 5 µm.

The observed ICCCs are spindle-shaped and resemble taste-bud sensory cells, plentifully equipping the skin of the zebrafish lips. The ICCC shape closely resembles that of gustatory receptor cells, but these differentiated epithelial sensory cells are not grouped into organs. The ICCCs that we found were elongated, with their deep pole immediately above the basal layer of the epidermis and an extended apical part reaching the surface of the epithelium. We found the nucleus at a basal position. The aforementioned cells are immunoreactive to BDNF antibody both in immunoperoxidase and immunofluorescent methods (Figure 8a–d).

## 3. Discussions

In teleosts such as zebrafish, the chemosensory system consists of taste buds and the olfactory epithelium [28,38]. The turnover and/or regeneration of zebrafish sensory epithelia, such as the potential role of neurotrophic factors including the BDNF/TrkB system, are well-known [10,39,40]. With aging, the senses of taste and smell significantly decrease [41,42,43], thus impacting life quality, changes in appetite or body weight, psychological wellbeing, and safety [44,45]. In daily human life, olfactory and gustative systems work together, so the loss or distortion of odors (dysosmia) and taste (dysgeusia) as hallucinations of taste or smell (fantogeusia, phantosmia) can be psychologically and physically debilitating both at home and at work [45]. Olfactory dysfunction occurs during the early stages of a series of neurological disorders, neurodegenerative diseases such as dementia, and mild cognitive conditioning [46], particularly Alzheimer’s disease and Parkinson’s disease [45]. Olfactory disorders can be caused by infections of the upper respiratory tract, inhalations of vapors [47], and general systemic pathologies [48]. Taste and smell disorders may occur because of head trauma, multiple sclerosis, and convulsive disturbances [46]. Loss of taste can occur after tonsillectomy [49]**,** and during the recent COVID-19 pandemic, the COVID-19 virus, and olfactory and gustatory dysfunction (OGD) were associated [50]. The sense of smell is fundamental in vertebrate life, as in zebrafish, to perform key functions to ensure survival [48,51,52]. Despite the morphological differences in the anatomy of the olfactory system among vertebrates, the neural basis of odor detection is highly conserved [53]. Environmental chemical information is transmitted from the olfactory organ to the brain, affecting alarm response, predator avoidance, food search, social communication, reproductive activity, and migration [54,55,56]. The olfactory system of teleosts can discriminate substances present in the water, and this information transmitted by the olfactory organ to the brain influences a behavioral response [54,55,56]. The regenerative abilities of olfactory epithelia both in zebrafish [57] and mammals [58] are known. BDNF is one of the growth factors involved in the generation and differentiation of new olfactive neuronal processes. BDNF transcription occurs in both the olfactory bulb and the olfactory epithelium during the regeneration process [59,60,61]. In the developing zebrafish brain, BDNF expression was observed in the nervous cells of the telencephalon, hypothalamus, spinal cord, mesencephalon, and thalamus, and in sensory organs including olfactory rosettes [15,58,62,63]. Moreover, BDNF was also found in the neurons of different anatomical districts of the adult zebrafish brain and sensory organs [12,15,64,65]. These findings agree with our results, where the BDNF was localized in the chemosensory cells of taste buds and in the ciliated sensory olfactory neurons in the sensory segment of the olfactory epithelium.

Calretinin was observed in the olfactory epithelium [53] and taste buds of different teleosts [33,66]. In zebrafish, a subset of olfactory sensory neurons was observed to be immunoreactive to calretinin [33,37,53,67,68]. Germanà et al. [11,33] showed the localization of protein S100 in olfactory crypt neurons. Our results showed that pro-BDNF, calretinin, and S100 colocalize in different subpopulations of olfactory ciliated cells of the olfactory epithelium of zebrafish larvae. In adult zebrafish, a subpopulation of ciliated cells in the sensory segment of olfactory lamellae showed BDNF, while the S100 protein was exclusively localized in the olfactory crypt neurons, as previously demonstrated by our investigation group [33,36,53]. Our data confirmed that, in the larval stage, the pro-BDNF precursor of mature BDNF is expressed, and in the olfactory epithelium, two calcium-binding proteins are expressed in two different types of sensory cells. Ciliated olfactory neurons detect bile salts, and microvillous cells detect amino acids [69,70]. In teleost fish such as zebrafish, taste buds are small pear- or onion-shaped intraepithelial sensory organs. Taste buds are composed of modified epithelial cells resting on a small dermal papilla [71,72]. The sensory epithelium of this organ is formed by elongated and vertically oriented cells: dark cells rich in microvilli, and light cells with long final microvillus. Basal cells described as Merkel-like basal cells are between sensory cells and the basal lamina [72]. The development and maintenance of these sensory organs are under the direct or indirect control of NTs and their Trk-like receptors [73,74,75]. Taste buds of adult zebrafish express TrkA- and TrkB-like immunoreactivity [11], as in mammals [76,77,78] and in *D. labrax* [79]. These data suggest that the sensory cells of adult zebrafish taste buds could be targets for neurotrophins [10] controlling different functions, especially regenerative processes [27,80,81,82]. Calretinin was localized in the taste buds and olfactory epithelium of different species of fish [67,83,84]. Our investigations demonstrate that the sensory cells of larvae and adult zebrafish taste buds show immunoreactivity for BDNF and calretinin.

Our data confirm the correlation between calcium-binding proteins and neurotrophins’ mode of action in sensory epithelia. Lastly, taste buds immunopositive to calretinin were observed in zebrafish larvae. In this larval stage, Calretinin immunopositivity is present only in the light cells identified by the presence of a single apical microvillus. In conclusion, the sense of taste and smell are fundamental in the life of vertebrates, including humans and teleosts. The regenerative abilities of the olfactory epithelia of zebrafish are known [85]. Moreover, the olfactory epithelium of mammals presents the characteristic of continuous neurogenesis throughout its life [58]. Taste buds are continuously added to the epithelium of the growing animal, so it is possible to observe early and mature taste buds both in the larva and adult zebrafish. Brain-derived neurotrophic factor (BDNF), a member of the neurotrophin family, and its signal-transducing Trk receptors (TrkB) play a crucial role in the development and maintenance of the nervous and sensory systems in mammals [2,3]. Calretinin, S100, and the other members of the CaBP family are involved in the control of the calcium balance from which important cellular functions depend (i.e., gene expression, synaptic transmission, cell cycle progression, and apoptosis). The reason for the difference in expression of Ca^2+^ BP by chemo and mechanosensory cells observed by Germanà et al. [33] could be explained by considering their innervation [86,87,88,89], embryonic origin [28,90], and function [33].

Further studies are needed to deepen the use of calcium-binding protein calretinin as a specific marker of ciliated cells of the olfactory epithelium and light cells of taste buds. Lastly, on the basis of the obtained results, we could assume that the zebrafish olfactory epithelium and taste buds represent an interesting model to investigate the involvement of growth factors in the chemosensory neurons that might be utilized in translational medicine. Studies are in progress in our laboratory targeted at creating mutant zebrafish for BDNF and TrkB to deeply analyze the functional activity of BDNF in the chemosensory organs of zebrafish.

## 4. Materials and Methods

In this study, we used larvae (5 days postfertilization) and adult zebrafish. The fish were obtained from CISS (Center of Experimental Ichthyiopathology of Sicily, University of Messina, Messina, Italy), the experimental protocols in this study followed the principles outlined in the Declaration of Helsinki, and samples used in this review coming from previous experimentation were approved by Italian Ministry of Health (A.M. n. 50, 8 August 2013). The fish at the above-mentioned stage were sacrificed with a lethal dose of tricaine methane sulfonate (MS222; 1000–10,000 mg L^−1^). The heads of adult zebrafish were quickly removed, fixed in 4% paraformaldehyde in phosphate-buffered saline (PBS) (AAJ19943K2, Thermo Scientific, Waltham, Massachusetts, Stati Uniti) 0.1 m (pH = 7.4) for 12–18 h, dehydrated through graded ethanol series, and clarified in xylene for paraffin wax embedding. Zebrafish larvae were fixed as a whole body and processed as above for paraffin wax embedding. Included tissue samples were then cut into 7 μm thick serial sections and collected on gelatin-coated microscope slides.

### 4.1. Trasmission Electron Microscopy

Samples were fixed by immersion in 2.5% glutaraldehyde in 0.1 M phosphate buffer (pH 7.4) at +4 °C, washed with 0.1 M phosphate buffer (pH 7.4), postfixed in 1% OsO_4_ in 0.2 M phosphate buffer (pH 7.4) at +4 °C for 1 h, dehydrated in graded ethanol, immersed in propylene oxide, and embedded in Durcupan (Sigma–Aldrich/Fluka, St. Louis, MO, USA). Ultrathin silver–golden sections were cut with a diamond knife on a Reichert Jung Ultracut E, placed on uncoated 200 mesh copper grids, contrasted with methanolic uranyl acetate, and lead citrate, and photographed with a JEOL JEM-100 SX transmission electron microscope at 80 kV.

### 4.2. Immunohistochemical Detection of BDNF, Pro-BDNF, Calretinin and S100 Protein in the Olfactory Epithelium, Taste Buds and ICCC

To analyze the expression of different proteins in the sensory patches of the olfactory lamellae, taste buds, and ICCC in zebrafish, serial sections were deparaffinized and rehydrated, washed in Tris–HCl buffer (0.05 M, pH 7.5) containing 0.1% bovine serum albumin and 0.2% Triton-X 100 and incubated in 0.3% H_2_O_2_ (PBS) solution for 3 min to prevent the activity of endogenous peroxidase; then, to rinsed sections was added fetal bovine serum (F7524 Sigma-Aldrich, St. Louis, Missouri, USA) for 30 min to avoid nonspecific binding, followed by incubation with the primary antibodies. Incubation was carried out overnight at 4 °C in a humid chamber with antibodies against pro-BDNF, BDNF, Calretinin, and S100 protein (see Table 1). These antibodies were characterized elsewhere in teleost and particularly in zebrafish immunohistochemistry by our group of research [12,19,22,24,25,91,92]. After rinsing in Tris–HCl buffer (0.05 M, pH 7.5) containing 0.1% bovine serum albumin and 0.2% Triton-X 100, the sections were incubated for 1 h at 4 °C with secondary fluorescent antibodies: antimouse IgG (H+L) Alexa Fluor 488, antirabbit IgG (H+L) Alexa Fluor 594, Anti Goat IgG (H+L) Alexa Fluor 594, Anti Rabbit IgG (H+L) Alexa Fluor 488 (see Table 2). Both steps were performed at room temperature in a dark humid chamber. Lastly, sections were washed, dehydrated, and mounted with Fluoromount Aqueous Mounting Medium (Sigma Aldrich, St. Louis, Missouri, USA). Sections were analyzed and images acquired using a Zeiss LSMDUO confocal laser scanning microscope with META module (Carl Zeiss MicroImaging GmbH, Jena, Germany) microscope LSM700 AxioObserver. Zen 2011 (LSM 700 Zeiss software, Jena, Germany) built in “colocalization view” was used to highlight the expression of both antibodies signals to produce a “colocalization” signal, the scatter plot, and fluorescent signal measurements. Each image was rapidly acquired to minimize photodegradation.

Moreover, to better analyze the morphological details of the positive cells, indirect peroxidase immunoreactions were carried out as follows: after rinsing in Tris-HCl buffer (0.05 M, pH 7.5) containing 0.1% bovine serum albumin and 0.2% Triton-X 100, the sections were then incubated overnight h at 4 °C with the antibody against BDNF (see Table 1), at room temperature in a dark, humid chamber. Thereafter, sections were rinsed in the same buffer and incubated with goat antirabbit IgG HRP-conjugated (see Table 2) for 1 h at room temperature. Lastly, sections were washed in Tris-HCl buffer (0.05 M, pH 7.5) containing 0.1% bovine serum albumin and 0.2% Triton-X 100, and the immunoreaction was visualized using 3–30-diaminobenzidine as a chromogen (manufacturer’s instructions, Sigma-Aldrich St. Louis, Missouri, USA, D5905). After rinsing in freshwater, sections were stained with Carazzi’s Hematoxylin Nuclear staining (05-M06012- Bio-Optica Milano S.p.A, Milano, Italy). Lastly, sections were washed in distilled water, dehydrated, covered with a coverslip using Eukitt mounting medium (09-00100- Bio-Optica Milano S.p.A, Milano, Italy), and examined under a Leica DMRB light microscope.

To provide negative controls, representative sections were incubated with specifically preabsorbed antisera as described above. Under these conditions, no positive immunostaining was observed (data not shown).

## Figures and Tables

**Table 1:** Primary antibodies.

Primary Antibodies	Supplier	Catalogue Number	Source	Diluition	Antibody ID
BDNF	Merck Millipore	AB1534SP	rabbit	1:100	AB_90748
pro-BDNF	Santa Cruz Biotechnology	sc-65513	mouse	1:100	AB_831028
Calretinin	Santa Cruz Biotechnology	sc-11644	goat	1:100	AB_634545
S100	Dako	Z0311	rabbit	1:100	AB_10013383

**Table 2:** Secondary antibodies.

Secondary Antibodies	Supplier	Catalogue Number	Source	Diluition	Antibody ID
Antirabbit IgG (H+L) Alexa Fluor 594	Invitrogen	A-21207	donkey	1:300	AB_141607
Antirabbit IgG (H+L) Alexa Fluor 488	Invitrogen	A-11008	goat	1:300	AB_143165
Antigoat IgG (H+L) Alexa Fluor 594	Invitrogen	A-11058	donkey	1:300	AB_2534105
Antimouse IgG (H+L) Alexa Fluor 488	Invitrogen	A-11001	goat	1:300	AB_2534069
Antirabbit IgG-peroxidase conjugate	Amersham	NA934	donkey	1:100	AB_772206

## Data Availability

All data presented this study are available from the corresponding author, upon responsible request.
